# 

**Published:** 1908-09-15

**Authors:** 


### ANNOUNCEMENTS.

NATIONAL DENTAL ASSOCIATION—OFFICERS FOR 1908-09—At the twelfth meeting of the National Dental Association, held in Boston, Mass., July 28th to 31st, 1908, the following officers were elected for the ensuing year: V. E. Turner, Raleigh, N. C., President; Wm. Crenshaw, Atlanta, Ga., Vice-president; for the south; Eugene H. Smith, Boston, Mass., Vice-president for the East; W. T. Chambers, Denver, Colo., Vice-president for the West; H. C. Brown, Columbus, Ohio, corresponding Secretary; Charles S. Butler, Buffalo, N. Y., Recording Secretary; A. R. Melendy, Knoxville, Tenn., Treasurer.

Executive Committee, new members, J. D. Patterson, Kansas City, Mo., C. J. Grieves, Baltimore, Md.; H. B. McFadden, Philadelphia, Pa.

Executive council: H. J. Burkhart, Chairman, Batavia, N. Y.; B. Holly Smith, Baltimore, Md.; F. O. Hetrick, Ottawa, Kan.; A. H. Peck, Chicago, Ills.; W. E. Boardman, Boston, Mass.

Birmingham, Ala., was selected as place for 1909 meeting.

H. C. BROWN, Cor. Sec'y,
185 East State St.,
Columbus, Ohio.

GILBERT E. CORBIN, M.D., D.D.S.—Doctor Corbin died at his home in St. Johns, Mich., August 6th, 1908, in his 77th year. Dr Corbin graduated from the medical department of the University of Michigan in 1855, and from the dental department in 1887. He was a man of the highest professional ideals and attainments. He practiced medicine then dentistry in St. Johns from 1862 to the time of his death. He was a man of high character and strict integrity and was a good citizen as well as a loyal professional man. He was for years a member of the State Examining Board of Registration for Dentistry and took great interest in the dental law. He was largely responsible for the enactment of the law that was repealed last winter. He leaves a widow and two sons, and many friends in the profession to mourn his death.

—✦—

PROCEEDINGS JAMESTOWN DENTAL CONVENTION.—We have just received from the committee in charge of its publication a copy of the proceedings of this convention. These proceedings were printed in the *Dental Cosmos* during last year and are put in form here as a permanent record of the details of that convention. They are nicely printed and contain several valuable contributions to our literature. Copies may be had by addressing Dr. M. F. Finley, 1928 I Street, N. W., Washington, D. C.

—✦—

DENTAL ORTHOPEDIA.—This is the title of a beautifully made book on the Correction of Dental and Facial Irregularities, by Calvin S. Case, M. D., D. D. S. Professor of Orthodontia in the Chicago College of Dental Surgery. It would seem like unmitigated assurance for us to undertake to review results of the life work of so capable and diligent a worker in this field of dental activity. Every reader is familiar with the great work Dr. Case has done for our profession in his frequent contributions to its literature and the successful practice of his art. His success in practice entitles him to authorship if not to recognition as a maker of standards of practice. To Case and Angle we owe largely the great progress that has been made in what is now known as modern orthodontia as a specialty. They have worked out for us practical methods for the treatment of these terrible deformities, and made the correction of the worst cases possible by the ordinary dental practitioner, to say nothing of the beautiful results accomplished by such as are willing to devote themselves exclusively to the practice of orthodontia. The book is beautifully made, it is printed on sized paper, with large type and is profusely illustrated. It could hardly be more elegantly done, and the bookmaker deserves great credit.

Dr. Case in his preface states that he makes the book for text use by students, and one finds his arrangement of the matter carries out this idea. The introductory chapter is devoted to his ideas of the proper methods of instruction and his nomenclature. He introduces some terms that are new and peculiarly of his own usage, and we suspect they will meet with some criticism. Some writers take occlusion as a basis for the correction of irregularities, while this author goes a step further and takes into consideration the facial or contiguous mal-developments, and applies his treatment with the idea of correcting both. Consequently he finds use for other names, or attaches other meanings to terms already in use. To this fact we can trace to a considerable extent the apparent differences between Dr. Case's nomenclature and principles of practice and those of other writers. We should then not be materially troubled by apparent conflicts of statements or seeming misuse of appliances or nomenclature. To such as have been brought up in the Angle School, Dr. Case's method of making his models from compound impressions instead of plaster, for instance, will think his method here wholly faulty. Dr. Case uses his models only as records of position of the teeth, while Angle makes them essential factors in diagnosis as well as permanent records, hence he requires a plaster impression which gives absolute details of teeth and alveolus. Dr. Case, on the other hand, requires a plaster cast of the entire side of the face to give him facial outlines for guidance in the changes of facial contours. After all it is the difference in view point as well as methods that largely influences our ideals, and it will be well for readers of this and other books on technical subjects to know something of their authors' aims, in order that they may comprehend their technical methods. We have'nt space to call attention to these differences in detail either in technique or nomenclature, but warn our readers that they will meet them at many points and may be annoyed by them. To one who will read this work with a proper conception of the authors ideals in view, he will be greatly pleased and edified.

The book is divided into *six parts*, each having several chapters devoted to the practical consideration of such topics as appropriately come under these several headings. The first part is devoted to the technique and treats of appliances and how to construct them. In order that Dr Case may carry out his methods he has gone to the trouble of providing all the appliances necessary to correct any case and these will be supplied from his laboratory at reasonable prices to any one who wishes to adopt his system of practice.

Part II, treats of the principles of orthopedia, mechanics of movement, anchorage, force and its application. Part III, diagnosis and treatment, typical and atypical occlusion, dento-facial relations, etc. Part IV, grouping or classifying malocclusions of the teeth and their arches. Part V, is devoted to the classification of dento-facial irregularities and the treatment. Dr Case makes seven classes:—1, mal-eruption of the cuspids with three types; 2, protrusions of the upper teeth with five types; 3, retrusion of lower teeth with normal uppers and four types; 4, retrusion of the upper teeth and five types; 5 and 6 include all types of maxillary protrusions and retrusions; 7, all open bite mal-occlusions. Part VI, is devoted to the principles and methods of retention. A large number of cases are illustrated and the treatments fully detailed, so that any one interested in this line of practice will be greatly entertained in following out the details. The work will, therefore, be of value and great benefit to the practitioner who occasionally does this work as well as the student and specialist.

General Anesthetics in Dentistry.—This is the title of a book of 300 pages issued by the John S. Noland Mfg. Co., of St. Louis, Mo., edited by William H. DeFord, A.M., M.D., D.D.S., of Des Moines, Iowa, formerly Professor of Oral Pathology and Surgery at the Iowa State University. The author has written his book in the form of a series of lectures intending to cover his subject step by step in a pedagogic manner, while his statements are, to a large extent, elemental; they are both sufficiently general as well as specific to allow his reader the option of preference in matters of practice. He illustrates by cases and experiences to such an extent as to give one the logical reasons for his practice and statements, and therefore eliminates the dogmatic which often provokes dissension or disbelief. The book is interesting reading and had we the space and time we should like to present some of the more important statements. Every dentist, whether he uses general anesthetics or not, should own and study this book, in order that he may be intelligent in the use or advisement of general anesthesia for the relief of dental pains.

The author presents the subject sequentially and systematically by chapters, and, as he speaks of them, lectures, beginning with a chapter on the legal and moral right of the dentist to administer anesthetics; then the value of them; to whom they should be administered; dangers; shock; fatigue; elements of success; relative safety; nitrous oxide; ethyl chloride; somnoform; chloroform; ether; then a couple of chapters on dangers, accidents, resuscitation, etc. It is a valuable contribution to our literature on the subject and should have a wide reading.

Principles and Practice of Filling Teeth With Porcelain.—Under the above title the Consolidated Dental Mfg. Co. has just issued a little book of 125 pages by Prof. John Q. Byram, of the Indiana Dental College. The book is the result of a series of papers on this subject contributed to the *Items of Interest* during last year. To those who have followed these writings no description may be necessary. The book takes up the subject largely from the technical aspect and in so doing necessarily gives the author's ideas as to the proper technical procedures. In the main these correspond largely with those of the best porcelain inlay workers. The author seems inclined to rest his practice more largely on mechanical helps in retention, rather more than some who believe in the principles of chemical and physical retention almost wholly. Some of the author's cavity preparations might be and doubtless will be criticised, as no two authors have yet been found to agree on any definite cavity preparation for metal fillings after many years of discussion. The author adds much of value to the color problem, which is a difficult one to many operators, and can never be worked out by any one into a definite rule of practice. He also gives us a lot of good data on the problem of fusing porcelain. It is a good book and every dentist should have a copy, as it will be a good thing to learn from and to work from or to as one feels inclined.

